# Sociocultural factors influencing decision-making related to fertility among the Kanuri tribe of north-eastern Nigeria

**DOI:** 10.4102/phcfm.v2i1.94

**Published:** 2010-05-07

**Authors:** Abdulkarim G. Mairiga, Abubakar A. Kullima, Babagana Bako, Mustapha A. Kolo

**Affiliations:** 1 Department of Obstetrics and Gynaecology, University of Maiduguri Teaching Hospital, Nigeria; 2 Centre for Arid Zone Studies, University of Maiduguri, Nigeria

**Keywords:** Africa, beliefs, contraception, couples, traditional family planning

## Abstract

**Background:**

The Kanuri tribe is found in the Lake Chad basin. However, the majority of the tribe lives in Borno State, Nigeria. Before this study was undertaken, factors related to fertility decisions among the tribe were not known.

**Objectives:**

This study is aimed at describing and documenting the sociocultural factors affecting decisions related to fertility among the Kanuri tribe.

**Method:**

The study applied the qualitative research method. In-depth interviews and focus-group discussions were used as data collection methods. Analysis was done manually.

**Results:**

Children among the Kanuri were highly valued and desired irrespective of their gender. The ideal family size, according to most of the respondents, was 16 children. Kanuri men are polygamous and can marry up to four wives in order to form large families. However, it is an abomination among Kanuri women to fall pregnant in quick succession; a phenomenon they termed konkomi. Other reasons for child-spacing were related to child welfare and maternal well-being. Methods for child-spacing included prolonged breastfeeding (*Nganji yaye*), ornaments in various forms and shapes, spiritual invocations and dried herbs (*Nganji Yandeye*). Few Kanuri women practiced modern methods of family planning.

**Conclusion:**

Trends in fertility among the Kanuri tribe need to be monitored regularly and appropriate measures be taken to introduce and promote modern family planning and child health services to ensure a healthier family life.

## INTRODUCTION

More than half a million women, nearly all of them in the developing world, die each year in pregnancy or childbirth. This amounts to one every minute.^1^ Another million suffer serious, sometimes permanent pregnancy-related injuries. Much of this suffering and death could be prevented through effective family planning engendered by modern contraception. Contraceptive use protects women from the health risk of unwanted pregnancies and gives women control over their lives.

The principal effort in population control is family planning, which aims at communicating to a society the desirability of limiting family size for economic, social and maternal health reasons.^2^ Family planning can be defined as a way of living that is adopted voluntarily upon the basis of knowledge, attitude and responsible decision-making by individuals or couples in order to pin the number, timing and spacing of the children that they want, so as to promote the health and welfare of the family group and contribute to the advancement of the society.^3^

Attempts to control population increase are as old as humankind himself. Evidence from medical history indicates that our forebears did space their children through traditional means and it has been observed that traditional methods of family planning were handed down either verbally or in writing from generation to generation as far back as the Stone Age.^4^ Before the introduction of modern methods, Africans had their own methods of fertility regulation. Nigerian culture includes many myths, rituals and the use of herbs in attempts to regulate women's fertility. Although many of these traditional methods of family planning have no harmful effects on a woman's health, some, however, do have dangerous or counterproductive effects.^5^ Nonetheless, the complete effectiveness of many of the traditional methods has remained doubtful,^5^ while modern methods of family planning and associated programmes have helped women worldwide to avoid 400 million unwanted pregnancies, saving many women from high-risk pregnancy or unsafe abortion.^5^

Modern family planning methods are widely believed to influence fertility worldwide.^6^ However, traditional methods are still used as the primary method of fertility regulation in African societies. The social, cultural and traditional beliefs and practices that are embedded in the social system have an impact on decisions related to fertility.^7^ In general, fertility patterns observed in developing countries can be attributed to the traditional attitudes and cultural values held by communities and the tradition of communities to favour having many children.^8^ Traditional family planning methods constitute a considerable proportion of the contraceptive methods used in both urban and rural Nigeria. The sociocultural factors that affect decisions regarding fertility and fertility regulation in north-eastern Nigeria are poorly understood.

Most people and governments recognise family planning as a basic human right, which necessitates the need for family planning programmes at all levels of health care services. However, in the early 1990s, family planning programmes faced the challenge of finding better ways to deliver services to millions of people who would use family planning. The behavioural changes demanded of the target population depend on a good understanding of the knowledge, attitudes and practices of individual towards family planning and child-spacing in the given community.

Research of fertility determinants during the last four decades has focused on economic and sociocultural factors that affect the attitude of individual or couples towards family size. The policies and programmes intended to bring about a change from large to small family norms cannot succeed without a thorough understanding of these factors in various socio-economic contexts; hence the need for this study cannot be overemphasised, especially in an environment with high fertility and low contraceptive use. This study was conducted in order to get some insight about fertility related issues among the Kanuri tribal group in north-eastern Nigeria. The objective of this study is thus to investigate the sociocultural factors that are involved in making decisions related to fertility and the nature of traditional contraception available in the Kanuri communities.

## METHOD

A qualitative study using key informants interviews and focus-group discussions (FGD) were conducted from December 2007 to January 2008 in two local government areas of Borno State, Nigeria. Saturation and redundancy of information were used to determine sample size. Key informants were recruited using heterogeneous and snowball sampling techniques. Interviews were tape-recorded. The interview and field notes were translated into English. Tape-recorded interviews were transcribed word-for-word. A contact summary note was written for each interview to summarize each encounter and to look for saturation and gaps. The transcribed and translated text document was entered into the Open Code Version 2.1 computer software for handling qualitative data and for coding and code sorting. Neutrality was maintained during coding and codes were categorised according to the major themes of the research question. Data were reduced to provide the researchers with an overall sense of the information that emerged under these themes.

### Study areas

The Kanuri tribe is found in the Lake Chad basin, occupying areas in Nigeria, Chad and Niger. However, the majority of the tribe lives in Borno State, Nigeria, which covers the greater part (69 436 km^2^ of the Chad Basin and is located in the north-eastern region of Nigeria. The capital of the state is Maiduguri, and the state population is estimated at 4 151 193 (64.37% rural, 35.63% urban).^9^ While English is the official language of communication, other major languages spoken in the area include Kanuri, Hausa and Babur/Bura. The inhabitants are predominantly Muslim.

Available statistics show that the reproductive health situation in this area is one of the worst in Nigeria.^10^ The crude birth rate was 43.60 per 1000, with gross fertility at 183 per 1000 and a maternal mortality ratio of 1549 per 100 000 live births.^10^ The National HIV/AIDS and reproductive health survey of 2003 found that only 2% of women in this region use modern contraceptives.^11^

For the purpose of this study, the Jere local government area (Jere LGA, urban) and Nganzai local government area (Nganzai LGA, rural) were selected by the team of researchers out of the eight local governments inhabited by the Kanuri in the Chad Basin area of the state.

### Site selection

This study was principally organised into urban and rural LGAs. One urban (Jere LGA) and one rural LGA (Nganzai LGA) were randomly selected. Within each urban and rural LGA, two study communities were also randomly selected: a reproductive health intervention site and a reproductive health non-intervention site. An intervention site is defined as a community, perhaps of ward level (with a population of more than 500 people), where a specific reproductive health (RH) intervention is carried out by a non-governmental organisation (NGO), or a public or private facility at the time of the research or in the very recent past (less than 3 months prior to the study period).

### Study design

This was a cross-sectional descriptive study. In this regard, multiple target groups were targeted in order to triangulate research findings and FGD, as well as key informant interviews, were conducted. In all, two different types of instruments were administered in each of the two LGAs selected. In each LGA two communities were randomly selected: a non intervention community and an intervention community. In each of the four communities, FGD and key informants interviews were conducted. The FGD targeted male and female youths between the ages of 15 and 35 years, as well as members of community-based organisations or NGOs, while the key informants interview targeted community stakeholders (e.g. religious leaders or teachers, traditional leaders, the wives of traditional leaders, medical personnel, women leaders and community development officers in the LGA). In each of the communities, the FGD consisted of five people as follows:

- FGD with five men (aged 15–35 years)
- FGD with five women (aged 15–35 years)
- FGD with five male members of civil society organisations
- FGD with five female members of civil society organisations
- Five key informant interviews with male community stakeholders
- Five key informant interviews with female community stakeholders.

In each of the FGDs and key informant interviews the following eight questions were asked, after collecting the respondents’ biodata:

1. In your opinion what is the ideal:
  - family size?
  - length of time between each child birth?
  - number of children you will like to have?
  - preferred sex of your child?
2. What are the advantages or disadvantages of having a large family?
3. What are the advantages or disadvantages of having a small family?
4. What do you think of when you hear the term family planning methods?
5. Are traditional or modern methods of family planning used in this community?
  - If so, what are the names of the traditional methods, what is their composition and when and how are they used?
6. What are your concerns about traditional and modern family planning methods?
  - What benefits do you see with using traditional versus modern family planning methods?
  - What disadvantages do you see with using traditional versus modern family planning methods?
7. Have you ever used a modern method of family planning?
  - If so probe to find out when, who administered and if still using it?
8. Have you ever used a traditional method of family planning?
  - If so probe to find out when, who administered and if still using it?

## RESULTS

### Characteristics of the study area and population

A total of 56 interviews were conducted; with a total of 120 respondents from all the communities. The ratio of men to women was 1:1. Most male respondents were polygamous. Only 38 of the respondents were educated to secondary school level.

### Perceptions and the need to regulate fertility

Children were highly valued and desired irrespective of their gender as both sexes fill a very crucial gap in the social and cultural life of a Kanuri family. The Kanuri people believe that the sex of the child does not matter because it is a gift from God and a blessing to the family. Couples with many children were respected; having many children was considered as insurance against the high child mortality prevalent in the area. The Kanuri also believed that children represented not only heritage of their descendants but were an asset for the parents at their old age. An elderly man from a rural non-intervention site said,

*‘Only three of my 12 children survived. Nine of them died due to illnesses during their childhood. I wish all were alive to take care of me at my old age time’*.

The desire to have many children was common in both the intervention and non-intervention communities. According to most of the respondents, the ideal family size was 16 children, but non-intervention communities tended to subscribe to an even larger family size. Kanuri men are polygamous and can marry up to four wives in order to form large families. A middle-aged man in a rural non-intervention site remarked, ‘*I have now six children from two wives. In order to have up to 40 children I have to marry up to four wives.’*

However, the desire to have many children was not directly translated to welcoming all births in close succession. It was an abomination among Kanuri women to have closely repeated pregnancies; a phenomenon they termed *konkomi* in Kanuri language. The Kanuri people thus allowed for 2–3 years between children in order to properly raise one child before the next is born. A middle-aged woman in a rural non-intervention site said,

*‘In Kanuri culture, we do not give birth to children one over the other in a row. If I have a child this harvest season, the next child would be after another two harvest seasons. I give birth only after the lasts child starts to walk and play by itself.’*

This cultural belief was common and strong in both the intervention and non-intervention communities. Child-spacing was strongly believed to help mothers regain their strength after delivery. Other reasons for this were related to child welfare; as lactating babies usually got sick and die when their mothers get pregnant before weaning them. There was also the cultural belief that successive and frequent pregnancies (*komkomi*) led to death of the lactating mothers or the infants or both of them.

In Kanuri communities, women first married when in their teens. Widespread practice of child-spacing did not allow women to have as many children as they would have liked, so, to offset this, many women in these communities continued have children well into their late-forties or even early fifties. Only menopause or ill-health prevented a woman from having children. Ill-health was another factor in taking the decision to use contraception.

### Traditional fertility regulation methods

Kanuri mothers breastfeed their children for two or more years. Non-practice of breastfeeding is believed to cause compromised child growth, ill-health and death. The advantage of breastfeeding for the prevention of pregnancy was well perceived and utilised in the Kanuri community. Prolonged breastfeeding (*Nganji Yaye*) in order to prevent pregnancy is widely practiced in both the intervention and non-intervention communities. Other methods for child-spacing among the Kanuri women included amulets in various forms and shapes (e.g. *guru*, *laya* etc.), which were tied to various part of the woman's body including the waist and arms and spiritual invocations on material items such as padlocks that were subsequently kept in the room or even under the bed of the couple. Other materials used included dried herbs (*Nganji Yandeye*) imported from afar. *Nganji Yandeye* was soaked in water and drunk by the woman after her menses. According to the Kanuri community, this would prevent the woman from getting pregnant till her next menses. Kanuri men were also fully involved in traditional child-spacing; in a FGD with men in one of the non-intervention sites, a man confessed on how he provided traditional contraception to his wife:

‘*There was a time when my wife had frequent pregnancies. Then, I got one traditional child spacing medicine from Saudi Arabia, Mecca called* Nganji Yandeye *in Kanuri. I put it in water and gave it to my wife to drink after her period has gone, since then she has been using it*.’

Few Kanuri women practiced modern methods of family planning, citing the opposition of their husbands, a fear of delay in return to fertility, damage to the reproductive systems (especially the uterus) and the belief that modern contraception was introduced to reduce the population of Muslim nations, as reasons for not using modern family planning methods. The key informants viewed the low patronage to modern contraceptives as a result of illiteracy, poor attitudes of health workers and the side-effects encountered by some women. These notions were reflected in both the intervention and non-intervention communities. However, men at the non-intervention communities principally attributed modern contraceptives to belonging to a foreign culture and causing infertility, while some claimed that they were not aware of modern contraceptive methods.

## DISCUSSION

The Kanuri tribe is polygamous, as is the case in many African communities, and one of the reasons for polygamy is to have many children. Teenage marriage is common among the Kanuri ethnic group. The age at which most Kanuri women first marry is 15 years, which is relatively young, when compared to the national median age of marriage, which is around 17 years for women.^11^ The reasons behind early marriage, according to other studies^12, 13^ in the region, include avoiding bringing ‘shame’ to the family through unwanted pregnancy, to reduce adultery and to prevent pre-marital sex.^12^ Also, an early marriage allows the women to grow in their marital homes as Islam permits.^13^ These practices are common to both intervention and non-intervention sites.

The desire to have many children in the Kanuri community is common among African communities^14, 15^ and evidently seen in the northern region of Nigeria.^11^ The 2003 National HIV/ AIDS and reproductive health survey^11^ indicated that the desire by the Kanuri to have these number of children is next only to the communities in the north-western region of the country, where 91% of respondents indicated their desire to have five or more children and that this decision was often left ‘up to God’ (that means as many as God wishes), as against 84% in Kanuri-dominated north-east and 51% in the south-west. This desire to have many children was found in both intervention and non-intervention communities, with possible little impact of intervention in the former communities. The desire to have many children among the Kanuri is related to the benefits children bring to the family, as is the case elsewhere in Africa.^8^ These benefits include respect in the community, a source of help and heritage for the family. The Kanuri welcome children irrespective of their gender, unlike the widely observed male preference in many societies.^16^ The gender-neutral preference is related to the belief among the Kanuri that children are gift from God and are thus a blessing to the family.

The desire to have many children is not directly translated to welcoming all births in close succession. Widespread practices of spacing do not allow women to have as many children as they want. Traditionally, the Kanuri women practice child-spacing in the way other women in tropical Africa do.^12^ This study has identified various factors that restrict the Kanuri couples from having the number of children they always desired. These factors include concern for child welfare, especially related to child feeding practices, as has been the case in Ethiopia and Bangladesh,^17, 18^ concern about the deterioration of maternal health due to closely spaced births, as in Ethiopia and Bangladesh^19^ and the cultural belief that successive and frequent pregnancies (*komkomi*) leads to death of the lactating mothers, the infants or both.

Kanuri couples widely practice traditional contraception methods to prevent pregnancy in ways similar to the tribal societies of India.^13^ Prolonged breastfeeding, known as *Ngaji yaye* among the Kanuri, is a factor that accounts for long birth intervals in tropical Africa.^20^ This method of contraception is more reliable and efficient than the more conservative, less reliable methods, such as ornaments (*guru, laya*), spiritual invocations and dried herbs. However, while the *Nganji Yandeye*, which are dried herbs imported from Saudi Arabia, might contain some active contraceptive ingredients, there is a need to identify and purify such active ingredients. The use of *Nganji Yandaye* or the practice of prolonged breastfeeding is a physiologic negative feedback mechanism on the hypothalamo-pituitary –ovarian axis that causes lactational amenorrhea. It is a form of natural family planning but its failure rate is high.

Factors affecting decision-making on fertility need to be monitored regularly and appropriate measures be taken to introduce and promote modern effective, efficient and safe contraceptive services to ensure a healthier life, especially in communities with a high prevalence of poverty, a desire for a large family size and poor girl-child education. As with the Kanuri, the acceptance of child-spacing can provide an advantage to promote modern contraceptives, which are more effective.

In conclusion, the Kanuri communities of north-eastern Nigeria have a strong desire for children irrespective of the gender. However, the desire for lager family size is restricted by the practice of child-spacing achieved by traditional family planning methods, which are not effective and sometimes dangerous to the health of the women. Low patronage for modern contraceptives was associated with both perceived and real threats. Intervention has had minimal impact, hence the need for more intervention which should address the perceived and real threats in the use of modern contraception. Information, education and communication also need to be intensified on the efficacy of modern contraception as opposed to the rampant use of traditional family planning.
